# The Use of Glass from Photovoltaic Panels at the End of Their Life Cycle in Cement Composites

**DOI:** 10.3390/ma14216655

**Published:** 2021-11-04

**Authors:** Kateřina Máčalová, Vojtěch Václavík, Tomáš Dvorský, Róbert Figmig, Jakub Charvát, Miloslav Lupták

**Affiliations:** 1 Department of Environmental Engineering, Faculty of Mining and Geology, VSB—Technical University of Ostrava, 17. Listopadu 15/2172, 708 00 Ostrava, Czech Republic; tomas.dvorsky@vsb.cz (T.D.); jakub.charvat@vsb.cz (J.C.); 2 Faculty of Civil Engineering, Institute of Environmental Engineering, Technical University of Kosice, Vysokoskolska 4, 04200 Kosice, Slovakia; robert.figmig@tuke.sk; 3 Faculty of Materials, Metallurgy and Recycling, Institute of Materials and Quality Engineering, Technical University of Kosice, 04200 Kosice, Slovakia; milo.luptak@tuke.sk

**Keywords:** photovoltaic glass, recycling, cement, raw material policy, cement composite

## Abstract

This article deals with the use of photovoltaic panels at the end of their life cycle in cement composites. Attention is focused on the properties of cement composite after 100% replacement of natural aggregate with recycled glass from photovoltaic panels. This goal of replacing natural filler sources with recycled glass is based on the updated policy of the Czech Republic concerning secondary raw materials for the period of 2019–2022, which aims to increase the self-sufficiency of the Czech Republic in raw materials by replacing primary sources with secondary raw materials. The policy also promotes the use of secondary raw materials as a tool to reduce the material and energy demands of industrial production and supports the innovations and development of a circular economy within business. The research has shown that it is possible to prepare cement composite based on recycled glass from solar panels, with compressive and flexural strength after 28 days exceeding 40 MPa and 4 MPa. Furthermore, a possible modification of the cement composite with different pigments has been confirmed, without disrupting the contact zone.

## 1. Introduction

Photovoltaic technology is one of the renewable energy sources with a relatively long lifespan, which is estimated at 30 years at least. As a great boom in this area took place at the end of the 20th century, we are entering the period of time when the expected end of life of the first photovoltaic panels is approaching and it is necessary to find a method to dispose of them after the end of their life cycle. Along with the rapid increase in the use of photovoltaic panels, there will be a proportionally increasing production of waste from solar energy. Only panels that were mechanically damaged due to improper handling during installation and transport have been disposed of so far [1,2].

Directive 2012/19/EU of the European Parliament and of the Council on waste from electrical and electronic equipment stipulates from 15 August 2018 that at least 85% of photovoltaic panel materials must be recovered and 80% of materials must be prepared for re-use and recycled [3]. There are 2 basic types of photovoltaic panels–silicon-based panels (monocrystalline, and polycrystalline from amorphous silicon) [4,5,6,7] and thin layers (thin layer types) [5,8]. A panel consists of a front layer–impact-resistant glass, an EVA layer (ethylene vinyl acetate), a solar cell placed below the EVA layer and a polyvinyl fluoride (PVF) back side or a combination of polyvinyl fluoride with polyethylene terephthalate (PTE). There is also a junction box on the back side, which serves as an output connection. A panel prepared in this way is also framed in aluminium profiles [9]. The recycling of solar panels at the end of their life cycle produces several components, namely 67% recycled glass, followed by aluminium 18%, plastics 11%, silicon 3% and metals 1%. [10]. Due to the high percentage of recycled glass as one of the components of recycled photovoltaic panels, our research is focused on the use of this glass.

The use of waste from photovoltaic panels as secondary raw materials and thus their recycling is considered due to the great benefits, which would include cost reduction of their disposal, environmental protection, and conservation of primary raw materials. A thorough understanding and reliable prediction of their effects is essential for their practical use as new construction materials.

The incorporation of photovoltaic waste (specifically glass from photovoltaic panels) into the cement matrix could be one of the new directions of possible recycling of waste materials from photovoltaic panels. New cement composites would be created and secondary raw materials would be used.

Waste glass can be used as a partial replacement for Portland cement in the amount of 10–30% of the weight. In the case of partial replacement of cement with waste glass in the form of glass powder, CO_2_ emissions are reduced, which helps to improve the environmental conditions and to reduce the amount of waste glass that would otherwise be landfilled [11]. Another option is to replace natural aggregate with photovoltaic glass in various fractions. In the concrete recipe, aggregate represents about 70%, leading to a greater use of waste glass [11,12]. One alternative to the use of waste glass is the production of aggregate from expanded glass (EGA), which is made from finely ground waste glass mixed with suitable expanding agents. [13]. Expanded glass aggregates could be used as a substitute for lightweight aggregates. These are light thermal insulation materials which can be used as thermal insulation cement composite. Expanded glass aggregates reduce the compressive strength of concrete because they have porous structure and lower mechanical strength. 100% replacement of aggregate with expanded glass aggregate can result in total carbonation [14]. Since waste glass is considered a pozzolanic material, it can be used as a partial replacement of cement in the production of ultra-high strength concrete (UHPC) [12]. Partial replacement of limestone filler with glass powder and blast furnace granular slag improves the mechanical strength of concrete. Compressive strength increased significantly between 28 and 90 days. Lower sorptivity values were measured and the gas permeability coefficient was lower as well [15]. Mixed colored waste glass was used as a partial replacement for fine natural aggregates in various ratios. Cut basalt fibres were added to the waste glass. The binder consisted of Portland cement and metakaolin (CC)—10% of cement weight [16]. The mechanical properties of the concrete treated with waste glass are affected by the amount and size of particles as well as the curing time. Concrete containing glass powder has lower strength during early aging. Its strength increases during late aging, compared to concrete which does not contain waste glass. Finer fractions of waste glass in a suitable ratio have a positive effect on the mechanical properties of concrete due to high pozzolanic reactivity and low alkali-silica reaction (ASR) [17].

## 2. Materials and Methods

### 2.1. Recyclate from Photovoltaic Panels

**Figure 1:**
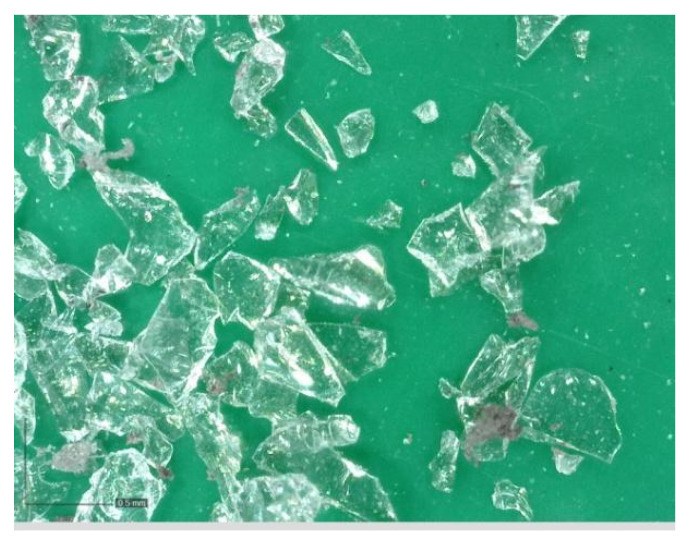
Glass recyclate fraction 0.0/0.5 mm; 103× magnified.

**Figure 2:**
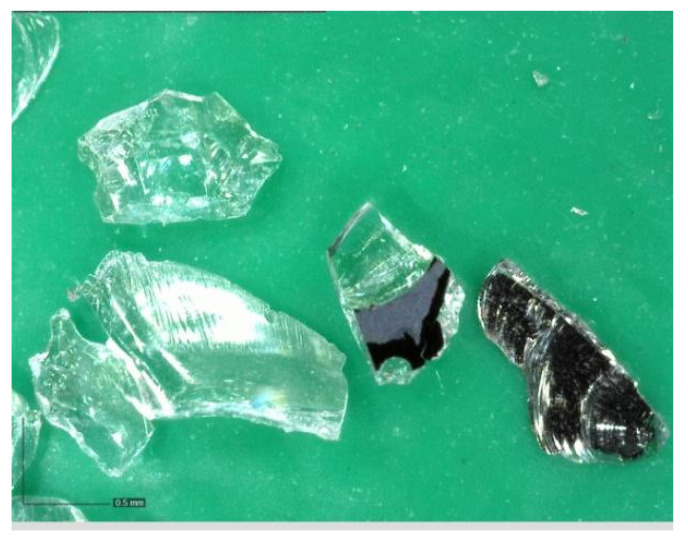
Glass recyclate fraction 0.5/1 mm; 103× magnified.

**Figure 3:**
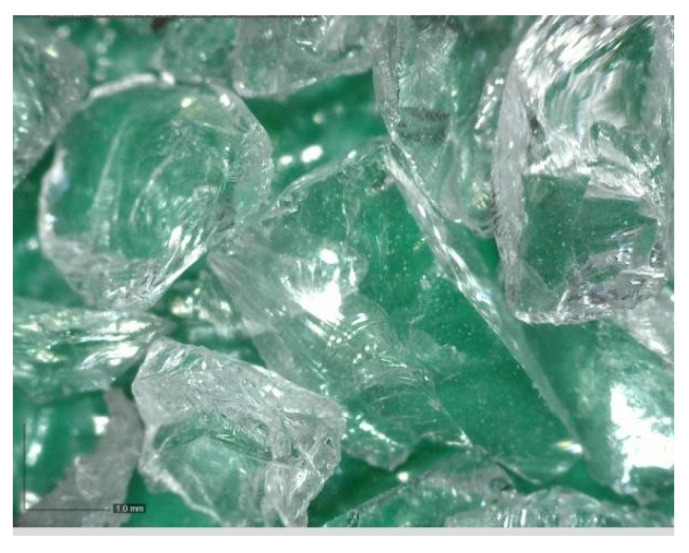
Glass recyclate fraction 1/4 mm; 51× magnified.

**Figure 4:**
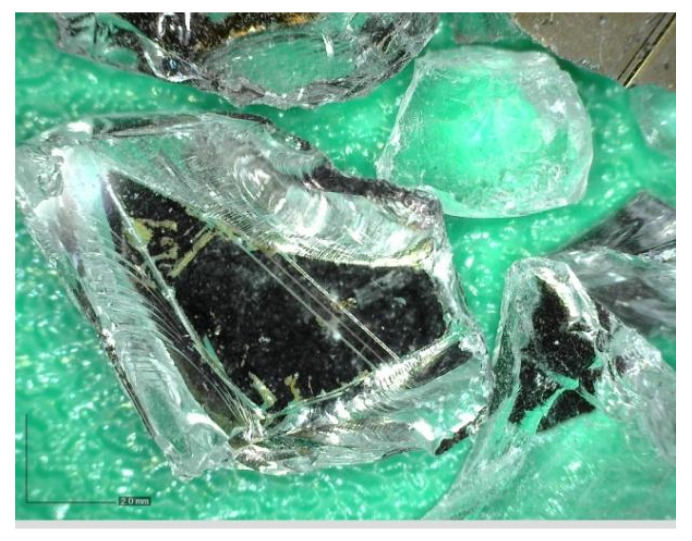
Glass recyclate fraction 4/10 mm; 51× magnified.

Photovoltaic waste glass (GR) was supplied by Bambas Elektroodpady Inc. company (Skalice n. Svitavou, Czech Republic) [18]. The glass was supplied in 4 different fractions: 0.0/0.5 mm; 0.5/1 mm; 1/4 mm and 4/10 mm. The glass was only crushed, no further treatments were applied. The photovoltaic glass was used as a 100% replacement for natural aggregate in the production of cement specimens. Figure 1, Figure 2, Figure 3 and Figure 4 show the photos of the individual photovoltaic glass fractions taken by an USB camera Dino-Lite (AnMo Electronics Corporation, Hsinchu, Taiwan) and magnified 103× (fraction 0.0/0.5 mm and fraction 0.5/1 mm) or 51× (fraction 1/4 mm and fraction 4/10 mm).

Recycled glass from photovoltaic panels of R3 recipe was subjected to chemical composition tests using the XRFS method. Tests of the extract of the cement composite prepared according to recipe R3 were performed afterwards. The results are presented in Table 1.

Table 1 clearly shows that the solidification of waste recycled glass from solar panels, which contains a high value of cadmium Cd, into the cement matrix is in the right way. Based on the results of extracts, it was found that the values of concentrations of the monitored analytes listed in Table 1 were reduced below the limit of detection by solidification.

### 2.2. Cement

Portland cement with the designation of EN 197-1-CEM I 52.5 R was used in the production of the test mixtures. The manufacturer is Cement Hranice (Hranice, Czech Republic), a joint-stock company. The production and requirements for cement are determined by the Czech technical standard EN 197-1:2012. The commercial name of the cement is TOPCEMENT. The following Table 2 shows the properties of Portland cement CEM I 52.5 R. One of the main properties of CEM I 52.5 R cement is its rapid increase in strength [19].

### 2.3. Mixing Water

Water from the water supply system was used in the production of the test mixtures. The criteria for the quality of mixing water are set out in the standard EN 1008–Mixing water for concrete–Specifications for sampling, testing and assessment of the suitability of water, including water obtained during recycling in a concrete plant, as mixing water for concrete [20].

### 2.4. Recipe Design

Using the determined optimal curves created in the 4C-Packing software [21] (Version 3.0, Danish Technological Institute, Taastrup, Denmark), a total of 5 recipes (R1 to R5) for the production of concrete mixture were designed. In these recipes, 100% of natural aggregate was replaced with recycled photovoltaic glass. The R0 recipe is a comparative one, in which the standardized aggregate was not replaced by recycled glass.

Recipe R0 was designed from Portland cement with the designation of EN 197-1-CEM I 52.5 R, mixing water from the water supply system and from standardized natural aggregates PG1, PG2 and PG3 [22] in the cement: water: aggregate ratio of 1:0.5:3. Recipes R1 to R5 were designed according to the same ratio as the R0 recipe, with the difference that 100% of natural aggregate was replaced with recycled glass from photovoltaic panels. The percentage representation of the individual photovoltaic glass fractions for the individual recipes R1 to R5 is presented in Table 3.

### 2.5. Preparation of Test Specimens

The test specimens were prepared according to recipes R1-R5 (see Table 3). The mixing was based on the standard procedure EN 196-1 [23] for the production of test specimens for testing the strength properties of cements. A laboratory mixer from BETON SYSTEM, BS MI-CM5AX, (Beton System Inc., Brno, Czech Republic) was used for the mixing. Mixing water and Portland cement CEM I 52.5 R were added to the vessel. The mixing started at low speed for 30 s. During the next 30 s, natural aggregate (recipe R0) or recycled glass was added in various ratios of individual fractions (recipe R1–R5 see Table 3). This was followed by rapid mixing for 30 s. The mixer was then stopped for 90 s so that the mortar could be wiped with a rubber spatula into the centre of the vessel during the first 30 s. This procedure was followed by rapid mixing for 60 s [23].

In the case of manual mixing (RM), the laboratory mixer was replaced by a PowerPlus mixer type POWE80070 (PowerPlus Inc., Vsetín, Czech Republic). The mixing speed and time were the same as the mixing speed and time in the laboratory mixer.

### 2.6. Methods Testing the Recyclate Properties

The following laboratory tests were performed on the recycled photovoltaic glass: determination of the geometric properties of the recycled glass according to EN 933-2 [24]. Density and water absorption tests were performed according to EN 1097-6 [25].

A sieve analysis of photovoltaic glass and natural standardized aggregates PG1, PG2 and PG3 was performed according to EN 933-2 standard on Testing of geometrical properties of aggregates—Part 2: Determination of grain size—Test sieves, nominal hole sizes. The standard provides the reference methods for determining the grain size of aggregates [24].

Density and absorptive capacity of the individual photovoltaic glass fractions were determined, including particles <0.063 mm, according to EN 1097-6—Testing of mechanical and physical properties of aggregates—Part 6: Determination of density and absorptive capacity of grains [25].

### 2.7. Metohods Testing the Cement Composites

The following laboratory tests were performed on the produced cement composites according to the proposed recipes (see Table 3) with 100% replacement of natural aggregate with recycled glass from photovoltaic panels.

The consistency of fresh mortar was determined using a jolt table including a metal cone according to EN 1015-3 for each produced cement mixture based on natural aggregate (recipe R0) and recycled glass (recipes R1 to R5) [26].

The strengths of the test cement composites were determined according to EN 196-1—Methods of testing cement—Part 1: Determination of strength [23].

### 2.8. Image Analysis

An image analysis of the samples of cement composites based on recycled glass from solar panels was performed using a DINO-LITE UNIVERSAL instrument (AnMo Electronics Corporation, Hsinchu, Taiwan).

### 2.9. Permeability

A rapid chloride ion penetration test (RCPT) according to ASTM C1202-19 Standard—Test Method for Electrical Indication of Concrete’s Ability to Resist Chloride Ion Penetration [27] was used to determine the permeability of cured composites. At the same time, the density was determined for each test specimen.

### 2.10. Test Specimens

The test specimens were beams measuring 40 × 40 × 160 mm according to [23] and 140 × 40 × 160 mm and a cylinder with a diameter and height of 40 × 40 mm. The moulds used for the production of the beams were filled with the cement mixture according to experimental recipes R1–R5 (see Table 4). The moulds were filled in two layers. Each layer was compacted using a compacting table (Siemens D-91056 Erlangen, Brio Hranice s.r.o., Hranice, Czech Republic). Subsequently, the excess layer of concrete was removed and the surface was smoothed horizontally with the surface of the mould using a trowel. A foil was laid on the treated surface to prevent water from evaporating from the concrete mixture and also to prevent the disruption of the concrete hydration process. The specimens were demoulded the next day and placed in a water bath at 20 ± 1 °C for 2, 7, 28, 90, 180 and 360 days. Test specimens with a diameter of 40 mm and a height of 40 mm at the age of 28 days were prepared to determine the permeability of the cement composite using the RCP test. They were provided with an impermeable coating along their circumference.

## 3. Results and Discussion

### 3.1. Density and Absorptive Capacity of Recycled Glass

The results of the determination of density and absorptive capacity of recycled glass are presented in Table 5.

Table 5 shows that the apparent density of the grains ρ_a_ is within the range of 2.46 to 2.49 Mg/m^3^. The density of the grains dried in the dryer ρ_rd_ is within the range of 2.45 to 2.49 Mg/m^3^. The density of the grains soaked and surface dried ρ_ssd_ is within the same range as the density of the grains dried in the dryer. The absorptive capacity after 24 h of immersion in water WA_24_ is within the range of 0.01 to 0.28%. The lowest absorptive capacity was 1/4 mm, namely 0.01 Mg/m^3^.

### 3.2. Grain Size Composition of Recycled Glass

**Figure 5:**
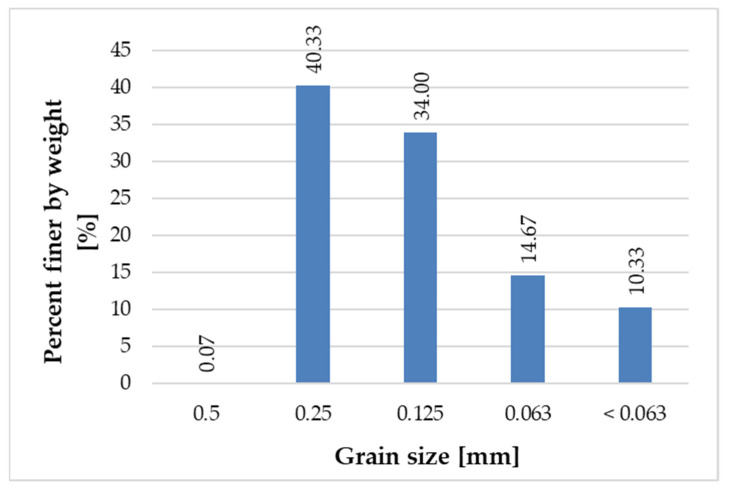
Grain size representation of recycled glass fraction 0.0/0.5 mm.

**Figure 6:**
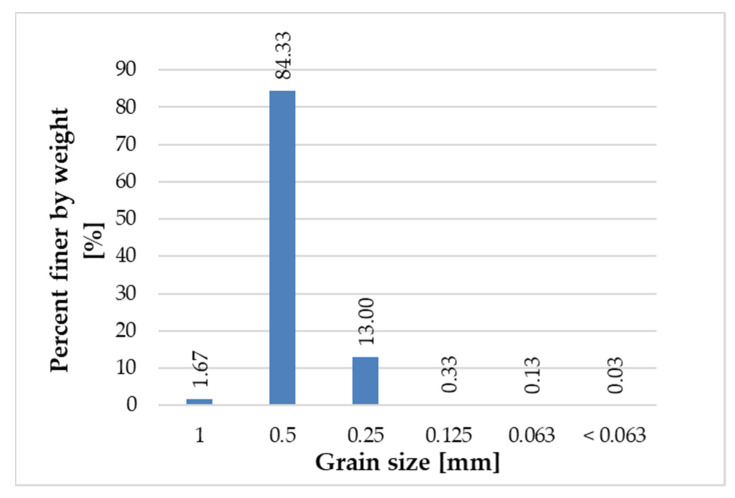
Grain size representation of recycled glass fraction 0.5/1 mm.

**Figure 7:**
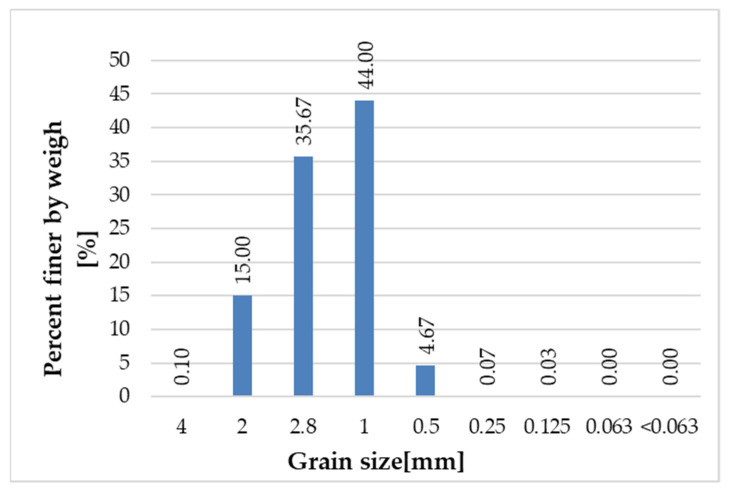
Grain size representation of recycled glass fraction 1/4 mm.

**Figure 8:**
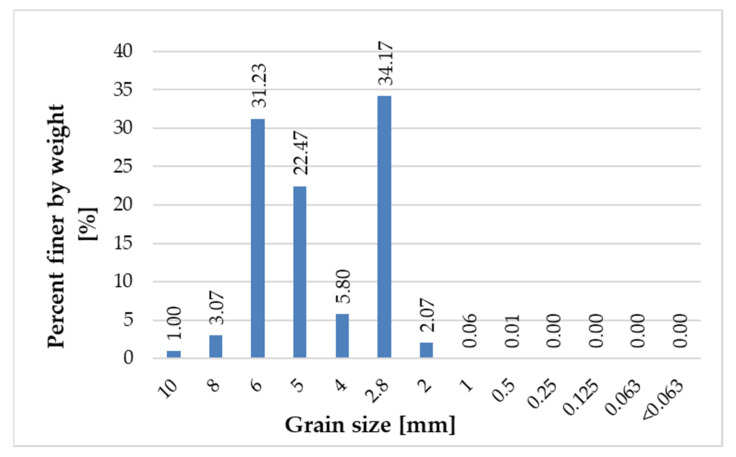
Grain size representation of recycled glass fraction 4/10 mm.

Figure 5, Figure 6, Figure 7 and Figure 8 present the grain size composition of recycled glass. The graph shows that the largest share of grains in the 0.0/0.5 mm fraction was represented by grains of 0.25 mm (approx. 40%) and grains of 0.125 mm (34%). In the 0.5/1.0 mm recycled fraction (see Figure 6), the largest share of grains was represented by 0.5 mm grains (approx. 84%). For the recycled fraction of 1.0/4.0 mm (see Figure 7) the share of grain size was as follows: grain size of 1 mm 44%, grain size of 2.8 mm 36% and grain size of 2 mm 15%. For the recycled fraction of 4/10 mm (it is a wider fraction) a significant share was formed by grains of 2.8 mm–about 34%, 5 mm–22% and 6 mm–31%.

### 3.3. Consistency of Fresh Concrete Mixture

The results of fresh cement mortar consistency tests are presented in Table 6.

The average spilling of fresh concrete mixed in the mixer is 190 mm; for hand-mixed concrete, it is 171 mm; and for concrete mixed in a mixer with pigment, it is 158 mm. The tests of the consistency of fresh cement mortar based on recycled glass have confirmed that the different percentage amounts of the individual fractions of recycled glass in the recipes do not affect the consistency of fresh mortar. Pigments have an effect on the consistency of fresh mortar. When using a pigment, it is necessary to take into account a decrease of the spillage value in comparison with fresh cement mixture without pigment by about 17%.

### 3.4. Flexural and Compressive Strength

**Figure 9:**
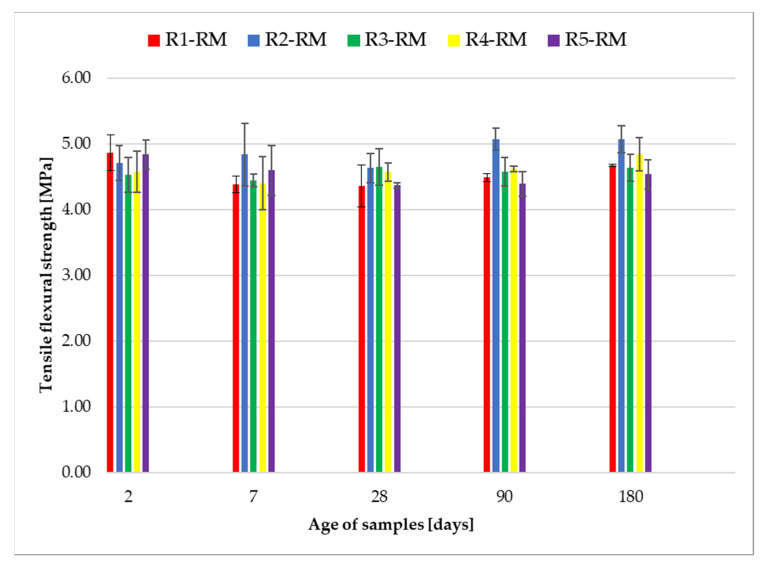
Graphic expression of flexural strengths after 2, 7, 28, 90 and 180 days.

Figure 9 shows the flexural strengths of samples R1-RM to R5-RM. The RM designation of the sample means that it was mixed by hand using a Powerplus mixer, type: POWE80070. The individual flexural strengths are given for samples that were stored in a water bath for 2, 7, 28, 90 and 180 days.

The values of the standard deviations of the measurements for the flexural strengths are presented in Table 7. The values of the standard deviations range from ±0.02 MPa to ±0.47 MPa.

Figure 9 indicates that the R2-RM recipe showed the highest increase in flexural strength during the tests. After 90 and 180 days, the flexural strength reached the values above 5 MPa, with minimal differences. All recipes show a significant increase in flexural strength after 2 days. This is due to the CEM I 52.5 R cement used, because this type of cement guarantees a very fast increase in strength. The lowest flexural strength after 2 days was reached by the R3-RM recipe. After 7 and 28 days, the lowest strength was found in the R1-RM recipe, and after 90 and 180 days in the R2-RM recipe. It can be seen in Table 7 that the different representation of recycled glass fractions in the experimental recipes does not cause significant differences in flexural strength. The values of standard deviations ranged from ±0.02 to ±0.47 MPa.

**Figure 10:**
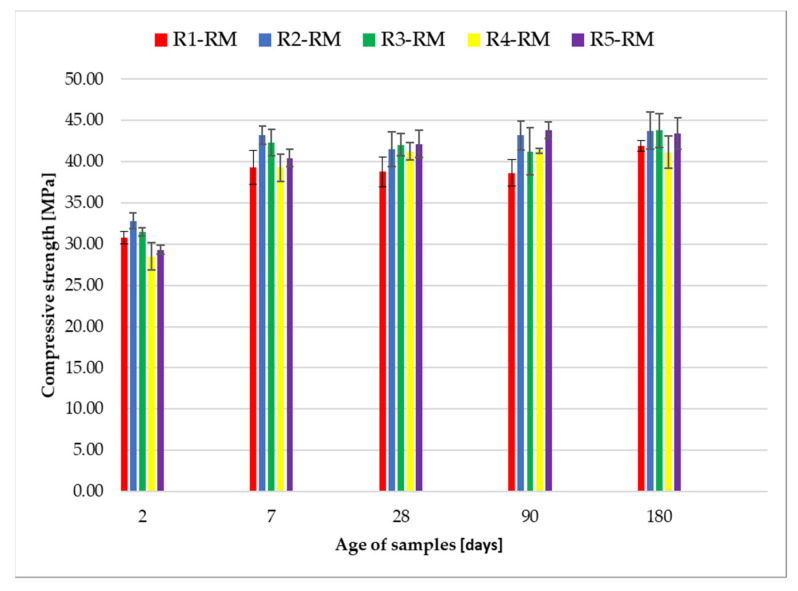
Graphic expression of compressive strength after 2, 7, 28, 90 and 180 days.

Figure 10 shows a graphical expression of compressive strength of the samples prepared according to recipes R1-RM to R5-RM. The individual samples were stored in a water bath for 2, 7, 28, 90 and 180 days. The graph shows higher initial compressive strengths after 2 and 7 days for all recipes, which is due to the use of CEM I 52.5 R cement. After 2 days, an average strength of approximately 31 MPa and after 7 days of approximately 41 MPa was achieved in all recipes. Furthermore, it can be seen that the strengths in the time interval of 7 to 180 days are similar. After 180 days, the average value of strength is about 42 MPa. Both flexural strength and compressive strength have confirmed that the different shares of recycled glass fractions in the experimental recipes (see Table 3) did not cause significant compressive strength differences.

The values of the standard deviations of the measurements for compressive strength ranged from ±0.26 to ±2.84 MPa (see Table 8).

**Figure 11:**
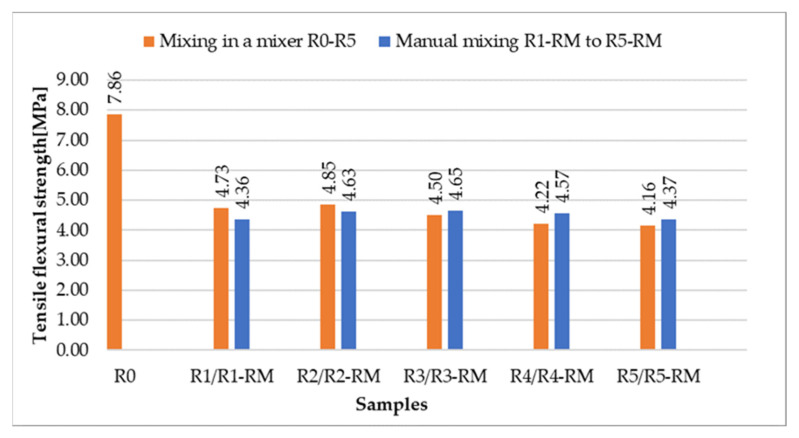
Comparison of flexural strengths after 28 days for samples mixed in a mixer and mixed manually.

**Figure 12:**
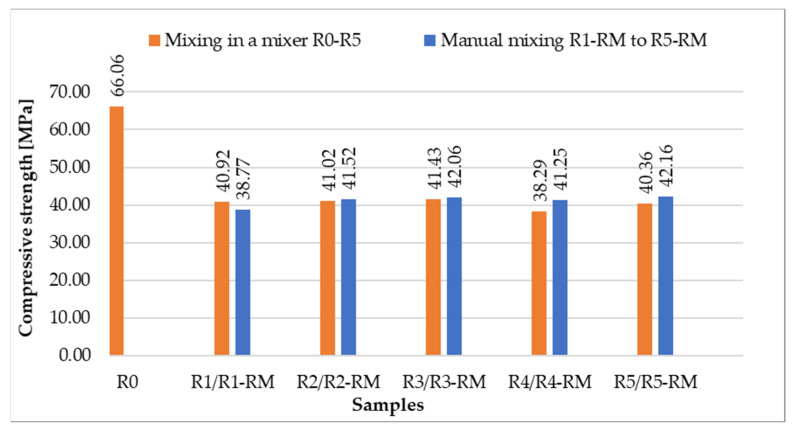
Comparison of compressive strengths after 28 days for samples mixed in a mixer and mixed manually.

Figure 11 and Figure 12 show the results of flexural strength and compressive strength in comparison with the comparative sample R0 with natural aggregate.

The test specimens were prepared in a laboratory mixer and with a hand mixer (see Section 2.5). The aim was to find out whether a different procedure of preparation of the test specimens will mean different values of the strength characteristics.

It is clear from the results that the flexural and compressive strengths after 28 days of the samples (prepared according to recipes R1 to R5), which were kept in a water bath, are almost identical for all the designed recipes. The strengths of the samples (recipes R1 to R5) in which natural aggregate was replaced with recycled glass (100% replacement) decreased by approximately 1/3 compared to the comparative sample R0. This significant decrease can be explained by the fact that the contact zone between the recycled glass grain and the cement paste is disrupted in the case of recycled glass composites. This is due to the smooth surface of the recycled glass grains (Figure 1, Figure 2, Figure 3 and Figure 4). Another factor blind the strength decrease is the different grain size composition of the recycled glass used for recipes R1 to R5 compared to the natural aggregate of recipe R0. The maximum grain size of recycled glass is 10 mm, the maximum grain size of natural aggregate (sand) is 4 mm. The composition of the grain size curve of natural aggregate fraction 0/4 mm is based on the standard composition according to EN 196-1 [23].

In comparison with the experiment [16], in which the control mixture and the mixture with glass and basalt fibres were compared, in our experiment, there was a decrease in flexural and compressive strength. The control mixture, which did not contain glass and basalt fibres, had the strength of 35 MPa. Cubic specimens measuring 100 × 100 × 100 mm were used for compressive strength measurement. Cylinders with a diameter of 100 mm and a height of 200 mm were used for tensile strength measurement. The content of the optimal replacement of natural aggregate with waste glass is 20%. The compressive strength increased by more than 4% and the tensile strength increased by 15% after 28 days. The use of basalt fibres contributes to the increase of strength [16].

Similar research with the replacement of natural aggregate with waste photovoltaic glass was carried out at the University of Brno. Another type of cement was used for the research, namely CEM II 32.5 R. An average compressive strength of about 11.7 MPa was achieved in this case. In the second recipe, where recycled glass and one fraction of aggregate were used, the average compressive strength was 21.8 MPa [28]. In our experimental recipes, an average value of flexural strength of 28 MPa and a compressive strength of 40 MPa were achieved after 28 days.

**Figure 13:**
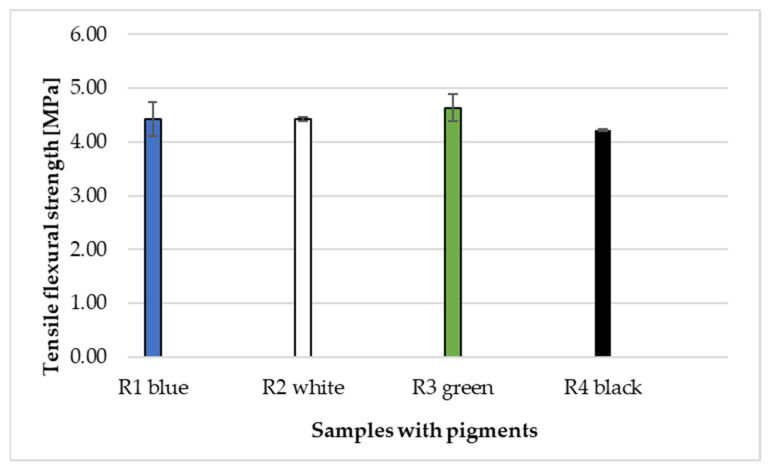
Flexural strengths of composites according to recipes R1–R4 with addend pigment.

The samples with added pigment (mixed in a laboratory mixer) were tested for flexural and compressive strengths after 280 days. Figure 13 presents the results of flexural strength.

In Figure 13, the highest strength after 280 days is reached by the cement composite made according to R3 recipe with added green pigment. The strength is 4.64 MPa. The samples made according to recipes R1 and R2 have the same strength. The sample made according to recipe R4 with black pigment has the lowest strength, namely 4.22 MPa. Standard deviations range from ±0.02 to ±0.31.

**Figure 14:**
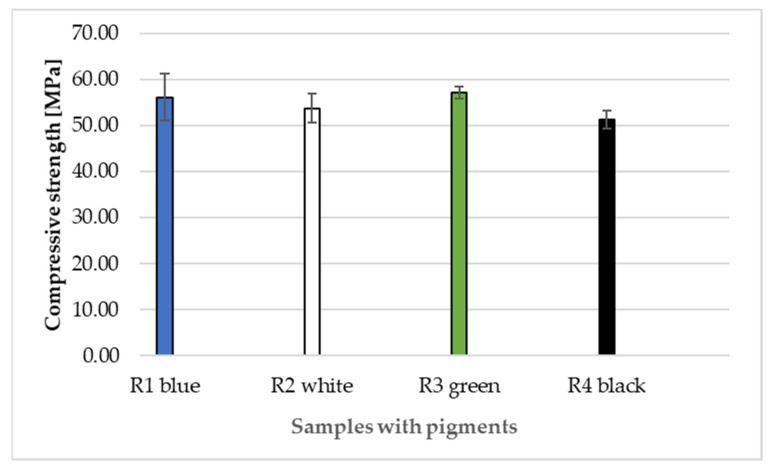
Compressive strengths of composites according to recipes R1–R4 with added pigment.

Figure 14 presents the compressive strength results of recipes R1–R4 with added pigment after 280 days.

Figure 14 clearly shows that the added pigment does not affect the compressive strength of the designed recipes. The lowest compressive strength is recorded in recipe R4 with black pigment, namely 51.32 MPa. This recipe also has the lowest flexural strength. Recipes R1 and R3 have similar strengths with a difference of 1 MPa.

### 3.5. Image Analysis

Two types of test specimen samples were prepared for the image analysis of cement composites based on recycled solar panel glass.

**Figure 15:**
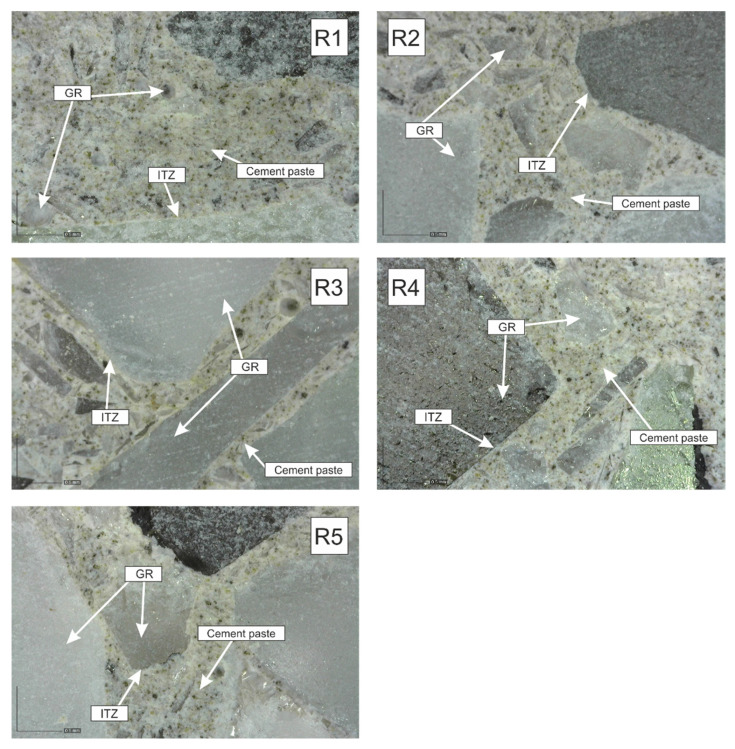
Image analysis of the cement composite samples according to recipes R1–R5; magnified 102×.

The samples from test specimens of experimental recipes R1 to R5 were the first type of sample used for the image analysis. They were not modified with a pigment and were not subjected to a polishing process. The results of the image analysis are presented in Figure 15.

In the other type, the samples for the image analysis were modified with pigments to enhance their structure. Beam-shaped samples were made according to the designed recipes R1 to R4. One color pigment was added to each recipe in the amount of 8% of the weight of cement (see Table 4). Iron Oxide Pigment (PRECHEZA a.s., Přerov, Czech Republic) [29] was used for the experiment. The designation of the pigments are–blue pigment–CB840, Ch. No. E1104; white pigment–R200M, Ch. No 162307; green pigment–G820, Ch. No. 900198; black pigment–B630, Ch. No 219785. Application of the pigments: recipe R1–blue pigment, recipe R2–white pigment, recipe R3–green pigment, recipe R4–black pigment. Figure 13 shows the texture of polished samples of cement composites with added pigments (recipes R1 to R4).

**Figure 16:**
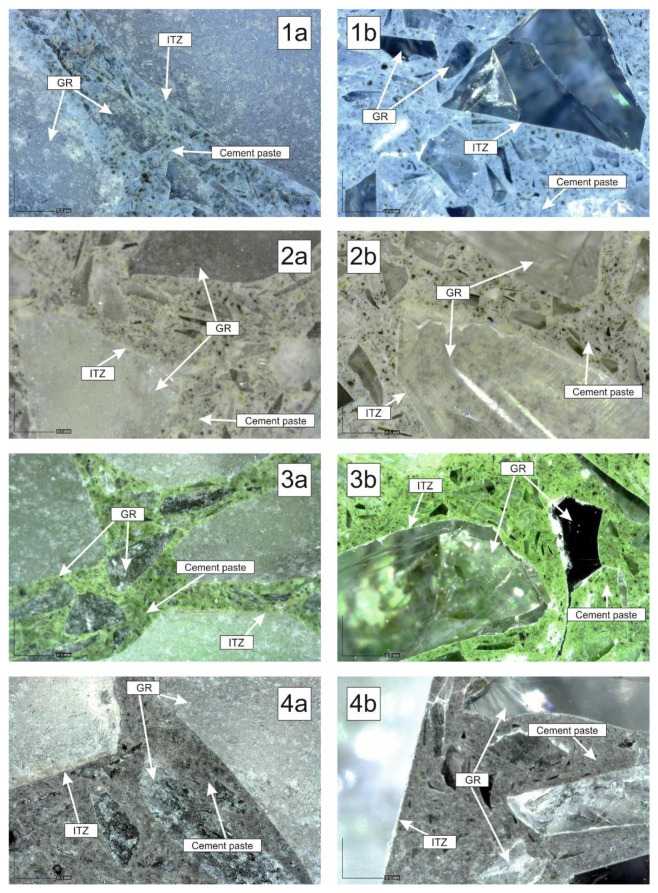
Structure of cement composites based on recycled glass with added pigment; (**a**)—unpolished composite; (**b**)—polished composite; 102× magnified.

Figure 16 shows the final composites with added pigment. The structure of the composite is magnified 102 times. The cement composites marked “a” are not polished. The composites marked “b” are polished using 220 mm, 400 mm and then 800 mm thick sandpaper. The sample polished in this way is finally polished on a cloth using a paste. The designation 1a-b in Figure 16(1a,1b) presents recipe R1 using a blue pigment, Figure 16(2a,2b) recipe R2 using a white pigment, Figure 16(3a,3b) recipe R3 using a green pigment, Figure 16(4a,4b) recipe R4 using a black pigment.

The following conclusions can be drawn from the image analysis results:

- The individual fractions of recycled glass from photovoltaic panels are evenly represented in the hardened cement composite. No effect of segregation of individual grains was found.;
- It can be seen that fine grains of recycled glass are evenly distributed in the cement paste;
- None of the samples (R1–R5) show disruption of the contact zone (ITZ) between the grains of recycled glass and the cement paste;
- It was confirmed that the cement composites based on recycled glass from solar panels can be polished and thus highlight the 3D effect of recycled glass grains fr. 4/10 mm.

### 3.6. Permeability

The permeability of hardened cement composites was determined on the basis of a standard 6-h RCP test at 60 V. The temperature of the electrolytes was checked during each measurement, especially the temperature of the test specimens, which ranged from 25 °C at the beginning of the test to 31 °C at the end of the test. With respect to this range, the effect of temperature on the charge transfer value can be neglected [30]. Table 9 shows the results of the measurements of density and total charge transfer of the individual test specimens.

**Figure 17:**
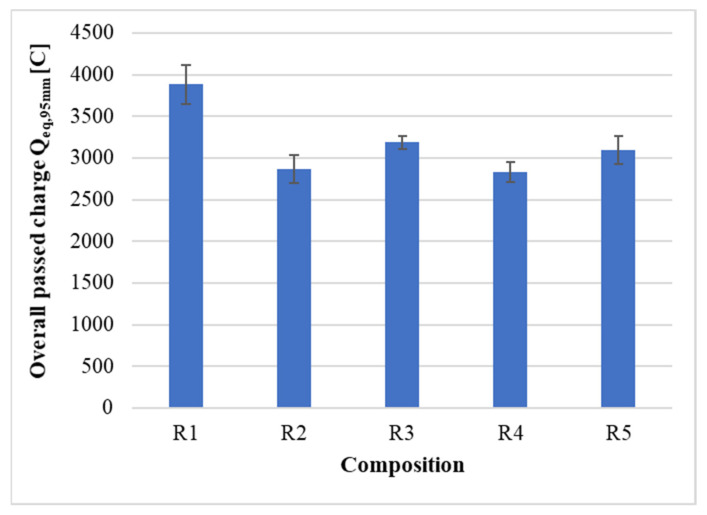
Results of the total charge transfer to the equivalent diameter of the test specimen.

After an analysis of the measurement results, the results R2-3 and R4-1 were discarded due to an apparent deviation, in which lower densities compared to the remaining test specimens resulted in an increase in the overall charge transfer. To exclude accidental error, the RCP test was repeated on these samples, but with the same result. No change was observed when repeating the test with the polished contact area on any of the test specimens of samples R1 to R5. In principle, there was no reason for that, as the sintering temperatures were not reached due to cooling and the polishing was carried out without any polishing agents. Figure 17 graphically shows the final values of the charge transfer Q_eq,95mm_ in the RCP test converted to the comparative diameter of the test body 95 mm, after taking into account the deviation of the results.

The durability of cement composites is the manifestation of the quantity and quality of the cement matrix. The quality is mainly influenced by the water-cement ratio [31], but also by the use of active admixtures [32]. Due to the same water-cement ratio and composition, the quality of the cement matrix within the individual samples R1 to R5 can be considered similar. The highest value of charge transfer is shown by the sample R1, 3884 ± 236 C, which corresponds to the lowest density of 2180 ± 5 kg·m^−3^. This may be caused by poorer compactibility and the formation of cavities/open micropores, which would indicate lower density and higher permeability [33]. Compared to the results of mechanical properties, the results are similar, and the sample of R1 also shows the worst properties. The best average results are achieved by the sample R4, 2833 ± 123 C with a corrected density of 2254 ± 4 kg·m^−3^. These results could be related to the highest content of 4/10 mm fraction. It would represent the smallest area of the interfacial zone, which is considered to be the most porous and thus the most permeable part of cement composites [34].

In general, all composites within the composition of R1 to R5 show “moderate chloride permeability (2000–4000 C) typical for conventional Portland cement concrete with w/c of 0.4–0.5 [27]”, which also corresponds to the designed water-cement ratio. The results are also comparable to a similar experiment using glass as a substitute for natural filler in concrete [35].

## 4. Results

Based on the current results of the research, the aim of which is a 100% replacement of natural aggregate in cement composites with recycled glass from solar panels at the end of their life cycle, the following conclusions can be drawn:

- Replacement of natural aggregate with recycled glass fraction 0/10 mm is possible;
- The densities of recycled glass fraction 0.0/0.5 mm, fraction 0.5/1 mm, fraction 1/4 mm and fraction 4/10 mm are similar and reach the values of approximately 2.5 mg/m^3^;
- The consistency of fresh cement mixture based on recycled glass was within the spillage range of 183–200 mm. This means that all the recipes were designed with a similar consistency of fresh cement mixture with natural aggregate and recycled glass from photovoltaic panels;
- The flexural and compressive strengths are almost identical for recipes R1 to R5. In case of flexural strength, the values are within the range of 4.2–4.9 MPa, in case of compressive strength, the values are within the range of 38.3–42.2 MPa;
- With 100% replacement of natural aggregate with recycled glass from photovoltaic panels in cement composites, it is necessary to take into account a decrease in flexural strength and compressive strength by approximately 20–30%;
- The results of the image analysis have confirmed the non-disruption of the contact zone between the grains of the recycled glass and the cementing compound. They have also confirmed the possibility of surface treatment of cement composites by grinding and polishing in order to enhance the 3D effect of glass grains in cement composite;
- Based on the RCP test, it has been found that the permeability of cement composites with recycled glass from photovoltaic panels shows values similar to conventional cement composites for a water-cement ratio of 0.4–0.5;
- The RCP test has also demonstrated the possibility of precise measuring and determination of permeability, and its further use in evaluating the internal structure of cement composites can be seen in the evaluation of the compaction, homogeneity or the potential of exposed surface of the cement composite to resist the penetration of aggressive substances;
- Future research will be focused on the modification of recipes R1 to R5 in order to increase the flexural strength to a minimum value of 6 MPa;
- The practical use of cement composites with 100% replacement of natural aggregate with recycled glass from photovoltaic panels can be: facing material for interior walls; construction of the upper layer of the floor (similar to teraso material);
- Future research will be focused on testing the alkali–silica reaction of recycled glass grains from solar panels. Furthermore, we will deal with the issue of surface treatment (grinding, polishing) of the designed cement composites for the purpose of their potential use in the interior as paving or tiling material.

## Figures and Tables

**Table 1:** Chemical composition of photovoltaic waste glass and cement composite extract.

Analyte	Crushed Mat. R3 Solid		Cement Composite Extract R3	
	Results [mg/kg]	Uncertainty [%]	Results [mg/L]	Uncertainty [%]
As	1.3	50	<0.001	-
Cd	186.0	30	<0.004	-
Cr	22.9	30	<0.010	-
Hg	<0.1		<0.001	-
Ni	15.8	40	<0.010	-
Pb	2.6	50	<0.020	-
V	2.2	50	-	-

**Table 2:** Portland cement properties CEM I 52.5 R [19].

Technical Parameter	Requirement of EN 197-1	Average Values Achieved
Compressive strength after 2 days [MPa]	≥30	42.0
Compressive strength after 28 days [MPa]	≥52.5	67.5
Beginning of setting [min.]	≥45	140
Volume stability [mm]	≤10	1.5
Sulphate cont. [% weight]	≤4.0	2.47
Insoluble residue [% weight]	≤5.0	0.29
Annealing loss [% weight]	≤5.0	2.55

**Table 3:** Percentage of the individual fractions of glass in recipes R1 to R5.

Formula	Recycled Glass Fraction [mm]			
	0.0/0.5	0.5/1	1/4	4/10
R1	13%	14%	0%	73%
R2	15%	14%	0%	71%
R3	14%	14%	5%	67%
R4	9%	8%	0%	83%
R5	9%	8%	9%	74%

**Table 4:** Recipe composition (dose in kg).

Components	R1	R2	R3	R4	R5
cement CEM I 52.5 R	563	563	563	563	563
water	281	281	281	281	281
GR fraction 0.0/0.5 mm	218	253	236	152	152
GR fraction 0.5/1 mm	236	236	236	135	135
GR fraction 1/4 mm	0	0	84	0	152
GR fraction 4/10 mm	1232	1198	1131	1401	1249
Water-cement ratio	0.5	0.5	0.5	0.5	0.5

**Table 5:** Determination of absorptive capacity and density of grains of individual recycled glass fractions.

	GR Fraction 0.0/0.5 mm	GR Fraction 0.5/1 mm	GR Fraction 1/4 mm	GR Fraction 4/10 mm
ρ_a_ [Mg/m^3^]	2.47	2.46	2.49	2.47
ρ_rd_ [Mg/m^3^]	2.46	2.45	2.49	2.45
ρ_ssd_ [Mg/m^3^]	2.46	2.45	2.49	2.46
WA_24_ [%]	0.28	0.14	0.01	0.25

ρ_a_—apparent density of the grains; ρ_rd_—density of grains dried in a dryer;.ρ_ssd_—density of soaked and surface dried grains;.WA_24_—absorptive capacity after 24 h of immersion in water.

**Table 6:** Determination of fresh mortar consistency by spilling test.

Consistency Ø [mm]	R0	R1	R2	R3	R4	R5
mixer mixing	185.0	187.5	183.0	199.5	185.0	198.0
manual mixing (RM)	-	184.0	165.5	177.5	158.5	167.0
mixer mixing with pigment	-	163.0	156.0	158.0	156.0	158.0

**Table 7:** Standard deviation values during the measurements of flexural strength [MPa].

Age of Samples [Days]	R1-RM [MPa]	R2-RM [MPa]	R3-RM [MPa]	R4-RM [MPa]	R5-RM [MPa]
2	±0.27	±0.27	±0.27	±0.31	±0.22
7	±0.13	±0.47	±0.10	±0.40	±0.38
28	±0.32	±0.22	±0.28	±0.14	±0.04
90	±0.06	±0.17	±0.22	±0.04	±0.18
180	±0.02	±0.20	±0.20	±0.25	±0.22

**Table 8:** Standard deviation values during the measurements of compressive strength [MPa].

Age of Samples [Days]	R1-RM [MPa]	R2-RM [MPa]	R3-RM [MPa]	R4-RM [MPa]	R5-RM [MPa]
2	±0.74	±0.95	±0.52	±1.66	±0.54
7	±2.07	±1.09	±1.58	±1.69	±1.02
28	±1.77	±2.13	±1.35	±1.06	±1.67
90	±1.58	±1.79	±2.84	±0.26	±0.97
180	±0.65	±2.25	±2.02	±1.91	±1.86

**Table 9:** Density D and total charge transfer Q.

Property	Unit	Specimen	R1	R2	R3	R4	R5
Density	kg/m^3^	1 2 3	2174 2182 2183	2212 2220 2186	2209 2209 2195	2218 2256 2251	2216 2225 2223
		avg std	2180 5	2206 18	2204 8	2242 21	2221 5
Overall passed charge	C	1 2 3	729 746 664	548 504 575	602 578 575	616 504 536	592 578 534
		avg std	713 43	542 36	585 15	552 58	568 30

## Data Availability

The data presented in this study are available upon request from the corresponding author.
